# Non-Chemical Control of Nymphal Longhorned Tick, *Haemaphysalis longicornis* Neumann 1901 (Acari: Ixodidae), Using Diatomaceous Earth

**DOI:** 10.3390/insects15110844

**Published:** 2024-10-28

**Authors:** Reuben A. Garshong, David Hidalgo, Loganathan Ponnusamy, David W. Watson, R. Michael Roe

**Affiliations:** 1 Department of Entomology and Plant Pathology, North Carolina State University, Raleigh, NC 27695, USA; rgarsho@ncsu.edu (R.A.G.); david.hidalgo@iniap.gob.ec (D.H.); wwatson@ncsu.edu (D.W.W.); 2 National Institute of Agricultural Research (INIAP), Quito 170518, Ecuador

**Keywords:** diatomaceous earth, *Haemaphysalis longicornis*, industrial minerals, longhorned ticks, non-chemical, tick control

## Abstract

The longhorned tick (LHT), *Haemaphysalis longicornis* Neumann (Acari: Ixodidae), is a serious invasive pest in North America where its geographical range is expanding with high densities associated with commercial animal production. There are only a few chemical pesticides available for LHT control, which can lead to the evolution of resistant strains. Diatomaceous earth (DE) was shown to be effective in killing some important tick species but was not examined for LHTs. When LHT nymphs were dipped for about 2–4 s into DE, transferred to Petri dishes (one tick/dish), and incubated at 30 °C and 70% relative humidity, the median survival time was 4.5 h. A locomotor activity assay showed that there was no difference in the overall distance traveled between the DE-treated and control ticks except during the first 2 h after exposure. In a field-simulated study in which a dose of 5.0 g DE/m^2^ was applied to pine needle litter infested with LHT, all the LHTs were dead at 24 h with no control mortality. Scanning electron micrographs showed the mineral adhering to all surfaces of the tick. The results indicated that DE is effective in killing nymphal LHTs and could be an alternative to the use of chemical acaricides with the advantage of managing pesticide resistance through the killing by a different mode of action and could be used for organically certified animal husbandry.

## Simple Summary

The longhorned tick (LHT), *Haemaphysalis longicornis*, is an invasive species of public health and veterinary importance. Having invaded North America, Australia, and New Zealand from East Asia, the LHT also has the potential to inhabit and survive in Africa, South America, and Europe. Synthetic chemicals have been vital in controlling these ticks but at the risk of the development of resistant strains and sometimes affecting non-target species. There is also a popular demand for non-chemical approaches for pest control. The use of diatomaceous earth (DE) derived from fossilized diatoms to control LHTs was not considered. This study examined whether DE could kill nymphal LHTs. When ticks were dipped into DE powder for a few seconds and then incubated at 30 °C and 70% relative humidity, they began dying as early as 2.5 h and were all dead by 9 h. The movement by walking of DE-treated nymphs was significantly higher in the first two hours and then the same as the control up to death. A dose of 5 g DE/m^2^ spread on pine leaf litter killed all the ticks. SEMs after treatment showed the mineral on large areas of the tick surface. These results indicated that DE has the potential of being used as a novel acaracide for LHTs.

## 1. Introduction

The longhorned tick (formerly Asian longhorned tick), *Haemaphysalis longicornis* Neumann [1] (Acari: Ixodidae), is an ixodid tick of veterinary and public health significance. The longhorned tick (LHT) is native to temperate East Asia [2], but its geographic range has expanded to include New Zealand, Australia, and, more recently, the United States [3,4,5,6]. There are also habitable regions in Europe, South America, and Africa where LHTs can survive [7]. The wide host range, parthenogenetic reproduction, and ability to complete its life cycle in an average time of three months facilitates the rate of spread of LHTs [2,4,5,8]. In the United States, the LHT is currently in 20 states since its first report outside of quarantine in 2017 in New Jersey, spreading at a rate close to three states per year [9]. If this spread continues across North America, predictive models suggest that the LHTs will be present in currently unoccupied states in the Pacific Northwest and in central and southern Mexico [10].

The LHT is of medical and veterinary importance as it is able to transmit *Theileria orientalis* complex and *Theileria mutans* Theiler [11], which cause a condition which can lead to severe anemia in cattle (theileriosis) and humans [5,7], tickborne encephalitis [12,13], and a Phlebovirus that causes severe fever with thrombocytopenia syndrome (SFTS) in humans [14]. Other pathogens found in *H. longicornis* whose transmission to other vertebrates remain unknown include *Rickettsia rickettsia* Ricketts [15], which causes Rocky Mountain spotted fever [16]; *Ehrlichia chaffeensis* Anderson et al. [17], which causes ehrlichiosis; *Anaplasma phagocytophilum* Dumler et al. [18] and *Anaplasma bovis* Donatien and Lestoquard [19], which cause anaplasmosis in humans and animals [20]; and *Babesia* spp., which causes babesiosis in animals and humans [21,22]. It is reported that, in endemic areas like China, the LHT plays a role in the epidemiology of *Borrelia burgdorferi* senso lato [23], but, in a laboratory study in the US by Breuner et al. [24], no *B. burgdorferi* transmission was observed. Furthermore, the LHT is reported to be associated with red meat allergy in China [25]. In the US, although the veterinary importance of the LHT is established [26,27], its ability to transmit pathogens to humans is still unknown [4]. However, Wormser et al. [28] and Rochlin et al. [29] warned us of the possibility that *H. longicornis* in US could transmit Heartland virus, a Phlebovirus that is closely related to that which causes STFS in endemic Asia. Additionally, bites from LHTs can cause erythematous pruritic lesions [30].

Due to the role that LHTs play in pathogen transmission, control is essential. Synthetic chemicals play a leading role in LHT control [31,32,33,34]. The use of non-synthetic products in LHT control are few [35,36,37,38]. The use of industrial minerals such as diatomaceous earth (DE) for LHT control is an unexplored research area. DE is a siliceous sedimentary rock comprising fossilized skeletons of diatoms that is considered environmentally friendly. DE has been shown to be acaricidal to some hematophagous ticks but has not been tested against LHTs [39,40,41,42]. The pesticidal power of any DE product depends on certain physical properties of its diatom particles such as the amorphous silicon dioxide content (which should be high), the uniformity of particle sizes (which should be less than 10 µm), its oil sorption capacity (which should be high), and the amount of impurities (which should be very little) [43].

We tested the acaricidal properties of a particular grade of DE called Celite 610 against LHT nymphs using a simple dipping bioassay and examined the treatment effects on locomotor activity and its coverage of the tick cuticle by scanning electron microscopy. We also conducted a proof of concept for the field use of DE for LHT nymph control by applying DE to pine needle litter infested with LHT nymphs. This study described evidence of the acaricidal potential of DE against nymphal LHTs.

## 2. Materials and Methods

### 2.1. Longhorned Ticks and Diatomaceous Earth

Live, unfed *Haemaphysalis longicornis* nymphs obtained in 2019 from a natural population in Roanoke, Virginia, USA were purchased from the Tick Rearing Facility at Oklahoma State University (Stillwater, OK, USA) under the USDA APHIS permit number 611-23-278-21434. In the laboratory at North Carolina State University, Raleigh, NC, the ticks were stored at 27 °C and 70% rh (14 h light: 10 h dark) for at least 24 h. The ticks used for the dipping and the locomotor activity bioassays were 75–85 d post-ecdysis, while those used in the simulated field studies were 29 d post ecdysis.

**Figure 1:**
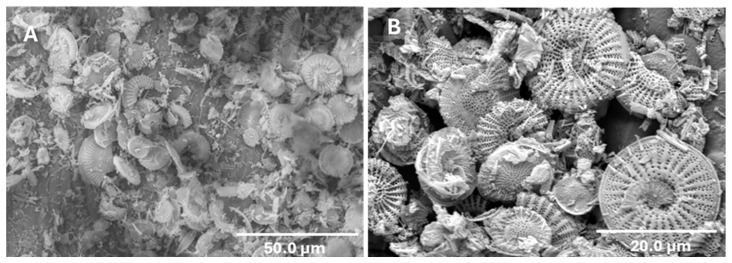
Scanning electron microscopy image showing the structure of the diatom skeletons that make up the diatomaceous earth that was used in this study at (**A**) a lower magnification of 50 µm and (**B**) a higher magnification of 20 µm.

The DE used was Celite 610 (Imerys Filtration Minerals, Inc., Roswell, GA, USA). Scanning electron microscopy (SEM) image of DE is shown in Figure 1. It was stored at room temperature in the dark in its original shipping container before use. All the bioassays were conducted in a Percival I-36NL temperature-humidity controlled incubator (Percival Scientific, Inc., Perry, IA, USA) at 30 ± 1 °C and 70 ± 5% rh (14 h light: 10 h dark).

### 2.2. Dry Dipping Bioassay

The nymphal LHTs were dipped in toto for about 2–4 s, one tick at a time, in 25 mg of DE in a Petri dish. To dip a tick, a clean #4 tip camelhair brush (Craft Smart, Irving, TX, USA) was used to pick the ticks and immerse them in the DE. Each dipped tick was transferred with the camelhair brush into a plastic Petri dish bottom measuring 55 mm in diameter and 13 mm in depth (Fisher Scientific, Hampton, NH, USA), capped, and sealed with Parafilm (Bemis Company, Neenah, WI, USA) at the edges to prevent tick escape. The same procedure was followed for the control ticks except that they were dipped in an empty plastic Petri dish using another clean (no DE) camelhair brush. In total, 30 nymphs were used in each replicate, 15 treated and 15 untreated. There were three replicates, each conducted on a different day. The control group was set up before the treatment to prevent contamination of the control with DE. Each Petri dish with ticks was placed into an opened plastic container (40 × 27 × 14 cm) lined with an exposed sticky tape all around the open edge, one for the treatment Petri dishes and one for the control, to serve as a second tick containment level. The nymphs were maintained during their normal photophase at 30 ± 1 °C and 70 ± 5% rh and inspected for mortality every 30 min.

Mortality was confirmed when a tick showed no sign of movement of any body part even after tapping the Petri dish several times. Dead ticks usually fold a few joints of their legs inwards toward their body [44]. To reconfirm that a tick was dead, the point of death was marked on the lid of the Petri dish and monitored over the next two observations, making sure that there was no change in position. Observation of tick movement was aided by a 4.3” 1000× lab handheld LCD digital microscope (Elikliv stores, Hillsdale, MI, USA).

### 2.3. Locomotor Bioassay

During the dipping bioassay, the ticks were monitored for locomotion activity. The initial central positions of the ticks in the plastic Petri dishes were marked as a starting point. Subsequently, at every 30 min inspection, the new positions of the ticks were marked using consecutive numbers. The distance between any two consecutive points served as the minimum distance moved by the tick within 30 min. Tick movement outside of the straight-line measurement would not be detected by this method. For every treatment dish, there was a corresponding control dish so that each control measurement was stopped after the death of its corresponding treated tick. Tick mortality was defined earlier. The minimum distance was measured up to the time taken for half of the treatment ticks to die (LT_50_). The mean of the distances moved in the treatment and control was used to determine whether there was a significant difference between the locomotor activity of treated and untreated nymphal LHTs.

### 2.4. Simulated Field Study

**Figure 2:**
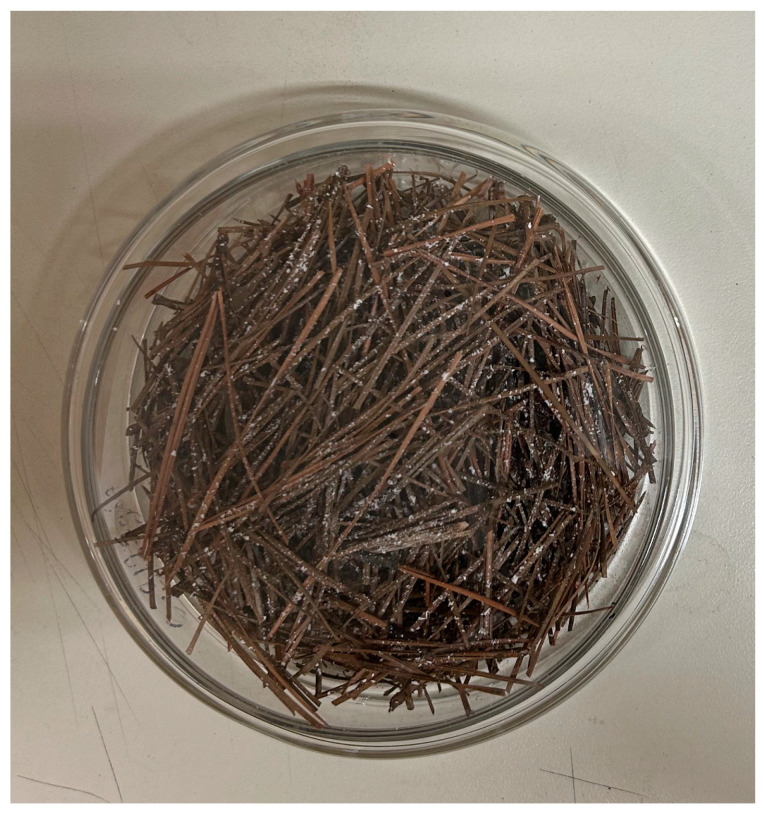
Five g/m^2^ of diatomaceous earth (white specks) spread on cut 8 g of loblolly pine, *Pinus taeda*, leaves in a glass Petri dish to simulate pine leaf litter in the field.

This study was conducted to determine whether the nymphs could be killed by DE when exposed indirectly by treating leaf litter. It also determined whether the nymphs of LHTs would freely move into DE-infested leaf litter when exposed to it in real-world scenarios. Dry, fallen, loblolly pine needles (*Pinus taeda*) collected at one time from the ground under the canopy of a natural secondary wooded habitat located at N35°47′13.43740′′, W78°41′55.26990′′ (near the Dearstyne Entomology Building of North Carolina State University, Raleigh, NC, USA) were cut into pieces averaging 5 cm in length (8 g) and placed into the bottom of each 120 × 20 mm glass Petri dish (Figure 2). The pine needles after transfer extended close to 2 cm above the bottom of the dish. Each Petri dish was infested with five LHT nymphs, one after the other, ensuring that the ticks were embedded in the leaf litter. A #4 tip camelhair brush was used to transfer each nymph into the pine needle leaf litter. Afterwards, 5 g DE/m^2^ was gently spread on the surface of the pine leaf litter by dipping a 1.9 flat camelhair brush into the DE and tapping the handle to spread it over the entire surface of the glass Petri dish (Figure 2). The Petri dish was then covered and sealed at the open sides with Parafilm to prevent the nymphs from escaping. The sealing process was carefully carried out to minimize shaking the dish. The control setup was without DE. Fifteen ticks (three glass Petri dishes) apiece were used for the control and treatment groups per replicate. Three replicates were conducted on three different days. The prepared plates were maintained at 30 ± 1 °C and 70 ± 5% rh for 24 h beginning from the normal tick photophase of 14:10 L:D. After 24 h, the ticks in the dish were inspected to determine how many were dead or alive by pouring each content of pine needle leaf litter on a white paper and carefully searching for the ticks, whether dead or alive. Tick death was judged based on whether the ticks moved when touched with a blunt probe and whether they were reactive to a short burst of breath from the mouth. Dead ticks were stored at −80 °C until needed.

### 2.5. Statistical Analysis

The data from the dipping bioassay were used to conduct a survival analysis curve using the ‘ggsurvfit’ (version 1.0.0), ‘survminer’ (version 0.4.9), and ‘survival’ (version 3.6–4) packages in the R programming software (version 4.3.3; R Core Team 2024, Vienna, Austria). Ticks that survived past the time at which the experiment was ended were considered censored. The time taken for half of the ticks to die (or the median survival probability) with associated 95% confidence limits was determined. Microsoft 365 Excel (version 2404) was used to plot the line and bar graphs associated with the locomotor assay. A *t*-test was used in the R program to determine significant differences in locomotor activity between the untreated and treated ticks and at two specific time intervals. The significance level was determined at an alpha value of 0.05 (or 5%).

### 2.6. Scanning Electron Microscopy (SEM)

SEM of dead *H. longicornis* nymphs was conducted from the 5 g DE/m^2^ treatment (n = 2 ticks) and compared to live (untreated) ticks (n = 2) from the simulated field study. The ticks used in the simulated filed study were placed into a plastic Petri dish, sealed, and stored at −80 °C until needed. SEM images were taken at the Analytical Instrumentation Facility at North Carolina State University. To begin, the ticks were mounted with super glue onto an aluminum Hitachi SEM mount. The tick samples were then vacuum-desiccated at 0% rh for 48 h and coated for approximately 1 min with a 70 nm gold–palladium mixture (60 Au/40 Pd) using a Cressington sputter coater to reduce charging effects. Afterwards, the ticks were scanned with a Hitachi SU3900 high-variable-pressure scanning electron microscope (Hitachi, Ltd., Chiyoda City, Tokyo, Japan).

## 3. Results

### 3.1. Dry Dipping Bioassay

**Figure 3:**
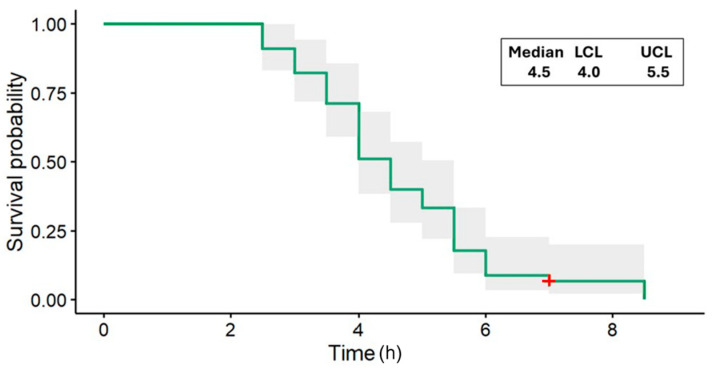
Longhorned tick nymph survival (survivor) function for dip assay estimated by the Kaplan–Meier method, including 95% confidence bands (shaded region) for 70 ± 5% rh (green). Censoring which occurred at 7 h is indicated by a red plus symbol. The estimated median survival times (at 0.5 survival probability) at 70% rh (and 95% confidence intervals at the median) are shown in the table insert. LCL, lower confidence limit; UCL, upper confidence limit.

All the *H. longicornis* nymphs (n = 45) that were dipped in DE were dead by 9 h. The LHT nymphs died as early as 2.5 h (Figure 3), recording an estimated median time of 50% survival of 4.5 h (95% confidence interval (CI) = 4.0–5.5 h). At 6 h, the estimated probability of survival of the *H. longicornis* nymphs dropped to approximately 0.10 (10%) (Figure 3). None of the 45 ticks in the control died during the timeframe of the dipping bioassay.

### 3.2. Locomotor Bioassay

**Figure 4:**
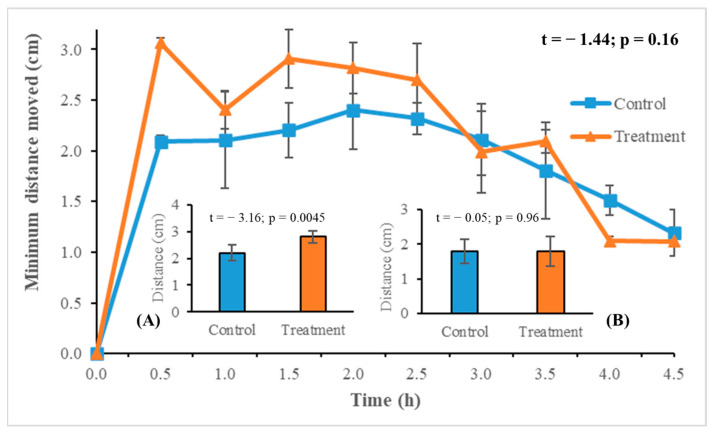
Longhorned tick nymph mean minimum distance (cm) moved at 30 min intervals for ticks dipped in diatomaceous earth versus the untreated control (line graph). The t- and *p*-value of the line graph is shown in bold in the upper right corner. The inserted bar graphs represent the mean minimum distances moved between (**A**) 0.5 to 2.0 h, and (**B**) 2.5 to 4.5 h (*t*-test, alpha = 0.05). Error bars represent one standard error of the mean.

There was no significant difference between the locomotor activity of LHTs in the control versus the treatment (t = −1.44; *p* = 0.16) at 30 ± 1 °C and 70 ± 5% rh (Figure 4). However, for the first two hours, the dipped LHT nymphs moved a greater distance than those in the control (Figure 4A; t = −3.16, *p* = 0.005). Afterwards, the movement of the ticks in both the treatment and the control decreased and did not show any significant difference (Figure 4B; t = −0.05, *p* = 0.96). On average, the ticks in the control moved 1.97 ± 0.47 cm compared to 2.43 ± 0.40 cm in the treatment but the overall difference in movement between the control and treatment was not significant (t = −1.44; *p* = 0.16).

### 3.3. Simulated Field Study and Scanning Electron Microscopy (SEM)

**Figure 5:**
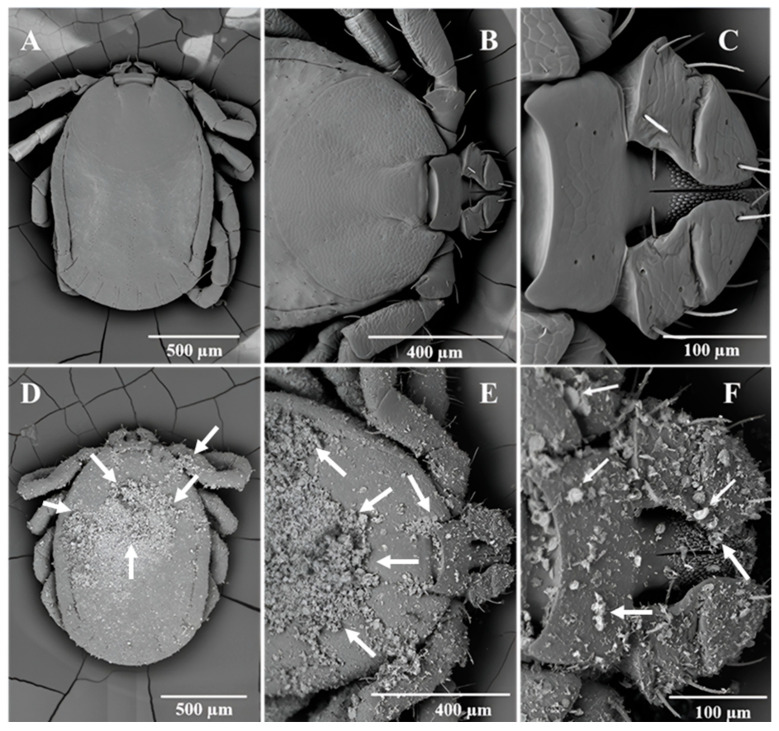
Scanning electron microscopy of (**A**) the entire dorsal view of the control showing the absence of diatomaceous earth (DE), (**B**) a closer view of the dorsum of the scutum and basis capitulum of the control, and (**C**) a closer view of the dorsum of the hypostome of a dead control longhorned tick (LHT) nymph retrieved from the simulated field assay after 24 h. (**D**–**F**) are dead LHT nymphs retrieved from the 5.0 g DE/m^2^ simulated field assay, each showing a similar orientation as the control image above it. The arrows are examples of DE.

**Figure 6:**
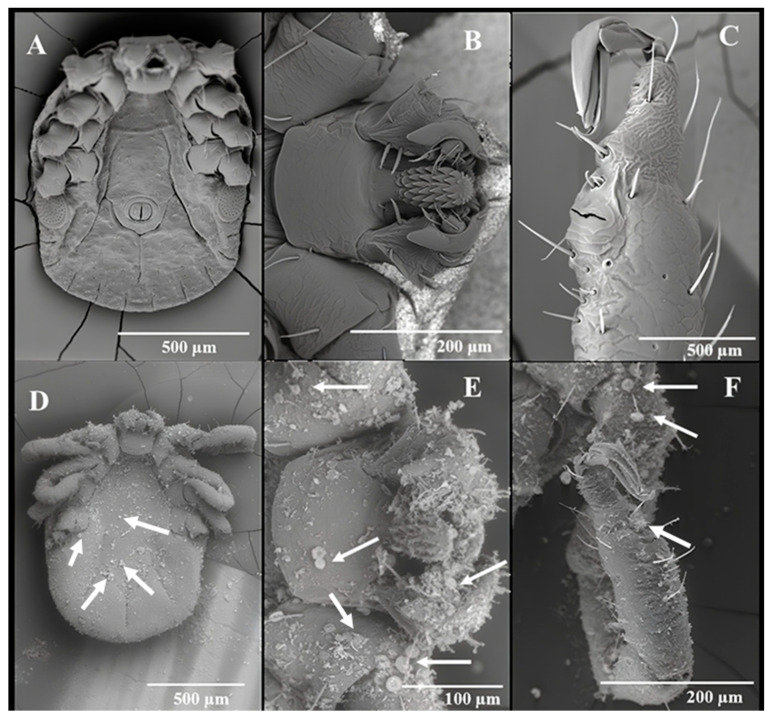
Scanning electron microscopy of (**A**) the entire ventral view of the control showing the absence of diatomaceous earth (DE), (**B**) a closer view of the ventral view of the basis capitulum of the control, and (**C**) a view of a leg of the dead control longhorned tick (LHT) nymph retrieved from the microcosm assay after 24 h. (**D**–**F**) were dead LHT nymphs retrieved from the 5.0 g DE/m^2^ simulated field assay, each showing a similar orientation as the control image above it. The arrows are examples of DE.

**Figure 7:**
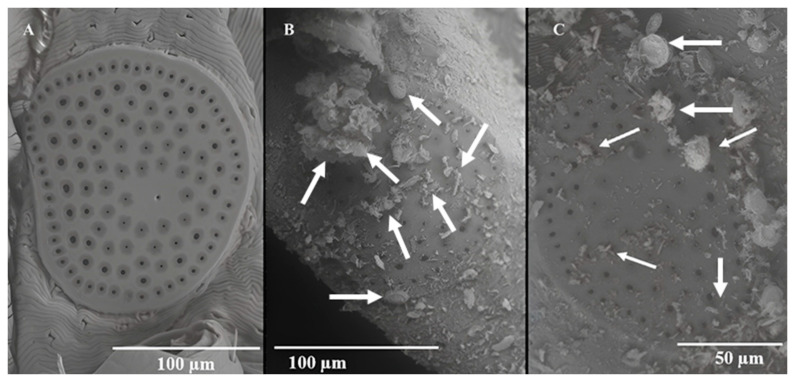
Scanning electron microscopy of (**A**) the spiracular plate of an untreated, (**B**) the spiracular plate of a treated, and (**C**) increased magnification of the spiracular plate of a treated longhorned tick nymph after 24 h in the simulated field bioassay (5.0 g DE/m^2^) that had died. The arrows are examples of diatomaceous earth.

By 24 h, all the 45 LHT nymphs in the treated pine needles containing 5 g DE/m^2^ had died. None of the control ticks (n = 45) died during the experiment. Figure 5A–C showed the dorsal view of a control (untreated) LHT under different magnifications and how they compared with LHT nymphs after 24 h in pine leaf litter treated with 5 g DE/m^2^ at 30° C and 70% rh. All the treated ticks were dead by 24 h and were heavily coated with DE (Figure 5D). The higher magnification of the treated tick showing the scutum and basis capitulum (Figure 5E,F) gave an indication of the mineral density on the tick surface. The arrows in Figure 5D–F point to different sizes (both full and bits) of fragmented diatoms on the cuticle surface on the dorsum. Figure 6 is the ventral surface of the untreated and treated tick, respectively, showing the whole body (Figure 6A,D), a magnification of the basis capitulum (Figure 6B,E), and the legs (Figure 6C,F) after the 24 h simulated field bioassay. The arrows in Figure 6D–F point to pieces of DE on different parts of the treated LHT nymph. Like the dorsal surface, the ventral surface of the treated nymphal LHTs were also covered with DE including the legs (Figure 6F). A closer observation of the SEM of the sieve (spiracular) plate of the DE-free (untreated) LHTs (Figure 7A) and the DE-coated (treated) nymphs of LHTs (Figure 7B; higher magnification: Figure 7C) showed pieces of DE scattered all around the sieve plate compared to the control. Some of the DE fragments seemed smaller than the spiracle pore size.

## 4. Discussion

The existing synthetic pesticides demonstrating acaricidal properties against LHTs (some in the lab and others in the field) include carbamates, organophosphates, pyrethroids, diamides, isoxazolines, formamidines, and insect growth regulators (IGRs) [45,46], with pyrethroids (this refers to both pyrethroids and synthetic pyrethroids which are derivatives of pyrethrins) listed as the most efficacious based on their residual activity [47]. Most non-pyrethroids either did not allow for control, did not provide 100% control, are not registered for use in the USA, or have been dropped from registration; the on-animal treatment emphasis in the US is mostly the use of pyrethroids to control LHTs (W. Watson, personal communication) [46]. Relying on a single mode of action like the pyrethroids is problematic because of the selection pressure for tick resistance. Efficient monitoring systems for resistance and understanding the mechanisms of resistance relative to their mode of action is beneficial for early detection [45,48], but the problem at this juncture is that there are limited acaricide alternatives with a different mode of action to use as a tool to manage pyrethroid resistance if it appears.

Embracing the idea of “One Health” (i.e., pesticides the public considers safe for humans, animals, and the environment), researchers have been exploring the use of botanical oils [49] and entomopathogenic organisms for LHT control [36,38]. For example, essential oils from the true cinnamon tree (*Cinnamomum verum*), Oregano (*Origanum vulgare*), and Mongolian thyme (*Thymus mongolicus*) were reported to disrupt fertility in engorged LHT females [50]. The Chinese cinnamon (*Cinnamomum cassia*) and the camphortree (*Cinnamomum camphora*) contain trans-cinnamaldehyde and diethyl phthalate, respectively, with the former being effective against unfed adults and the latter effective against immature LHTs [51]. Essential oils from lemongrass (*Cymbopogon citratus*) also killed nymphs and adult LHTs [35]. Arthropod pests do not easily become resistant to botanical oils since they comprise multiple actives [37,45]. In another biological approach, Lee et al. [36] tested fungi from the genera *Beauveria*, *Metarhizium*, *Cordyceps*, and *Akanthomyces* against LHTs and found isolates of *M. anisopliae* s.l. and *C. fumosorosea* to be effective against more than 50% of the unfed LHTs. *Metarhizium anisopliae* Metchnikoff [52] was ranked highest because of its superior virulence. *Metarhizium anisopliae* was reported to kill LHTs by secreting hydrolytic and lipolytic enzymes (proteases, chitinases, esterases, and lipases) that dissolve the cuticle of the tick, allowing the hyphae of *M. anisopliae* to germinate [36,53]. The organics and fungistatic agents (hydrocarbons) transported from the tick epidermis into the cuticle determines whether a particular tick species will be susceptible to a specific entomopathogenic organism [54]. Anti-tick vaccines are at an early stage of exploration for controlling LHTs [55,56].

One approach not investigated was the use of DE to control LHTs. DE is the fossilized remains of diatoms (mainly composed of amorphous silicon oxide) deposited during the Eocene–Miocene period that have undergone compaction and cementation to form sedimentary rock. As a powder, DE is well-known to have insecticidal activity against a variety of pests, mostly of agriculture and urban importance [57,58,59,60]. Most recently, Deguenon et al. [61], using a modified WHO funnel test, found a mimic of DE, milled expanded perlite, to be fast acting in killing the malaria mosquito, *Anopheles gambiae* Giles [62], even under high humidity conditions. Since mechanical insecticides like DE kill by dehydration [43], its efficacy under high humidity conditions like those found in some areas of Sub-Saharan Africa was not expected. Using the same assay, Chen et al. [63] found milled perlite was also active at high humidity against three species of filth flies. In the Phase II WHO studies when the same material was sprayed in water at the rate of 5.0 g/m^2^ on the inside walls of experimental huts with human subjects sleeping inside every night, the malaria mosquito was controlled based on WHO standards for six months while pyrethroids failed in one week [64]. Richardson et al. [40] found that both perlite and DE were active against ticks in lab bioassays and at high humidity. Showler and Harlien [42] found DE to be effective against *Rhipicephalus* (*Boophilus*) *microti* larvae in the lab and on cattle, while Showler et al. [41] found DE to be effective against *Amblyomma americanum* Linnaeus [65] larvae and nymphs. This discovery resulted in a renewed interest in the use of the mechanical insecticide, DE, for the control of arthropods of medical and veterinary importance like ticks, since DE already had a US EPA registration for insect control. The current view is that the DE mode of action in insects is the adsorption of wax (lipids) on the surface of the cuticle [66,67] and cuticular abrasion [67], resulting in dehydration and death.

Considering the limited options for the chemical control of LHTs and the rapid expansion of this invasive tick in North America, an examination of DE for the control of LHTs was conducted in this current paper. We targeted nymphs because, in the United States, nymphs of *H. longicornis* are the only life stage that persist all year round [68], which makes the nymphal stage a convenient stage to target when trying to control LHTs. The nymphs are also known to feed on livestock and are small enough to go unnoticed on the skin of humans compared to adults. In addition, unfed *H. longicornis* nymphs are the most dehydration-resistant life stage unlike other ticks which have their dehydration resistance decreasing from larva to adult [5]. In the dry dipping bioassay, DE began killing unfed nymphs of LHTs in as little as 2.5 h and reached a time to 50% mortality at 4.5 h (CI 4.0–5.5 h). The rapid mortality questions the currently accepted mode of action of cuticular abrasion for DE. A previous study which examined the dose response of DE on *Dermacentor variablis* Say [69] nymphs showed that DE was causing death in a dose as low as 0.15 g/m^2^. Scanning electron microscopy images of dead ticks at 0.15 g/m^2^ showed that there was almost no DE on the dorsal and ventral cuticle surfaces and no sign of abrasion, suggesting that it was not cuticle abrasion that was killing these ticks [70]. Additionally, if dehydration is the mode of action, then increasing the relative humidity should decrease the killing properties of DE [71] as was found for mosquitoes [61] and filth flies [63]. However, for the deer tick, *Ixodes scapularis* Say [69], the nymphs died faster at 70% rh (the time to 50% mortality (LT_50_) = 43.85 min (95% CI: 40.61–46.93)) than at 50% rh (LT_50_ = 81.68 min (95% CI: 77.44–85.86) [40]. A similar result was observed for unfed American dog tick, *Dermacentor variabilis,* nymphs [70]. Ticks begin secreting hygroscopic fluid from their salivary glands to absorb moisture from the air at a relative humidity of above 65% [72]. At a relative humidity of below 65%, ticks are more likely to alter their physiological behavior to conserve water [73]. Thus, at 70% rh, the ticks may attempt to secrete hygroscopic fluid, which results in more water loss and, eventually, death [70]. DE increased the LHT locomotor activity during the first two hours after exposure, suggesting that DE elicited a behavioral response. The mechanism that produces this LHT response and whether this is a factor in mortality are unknown.

Increased locomotor activity was also shown in unfed adult deer ticks, *I. scapularis,* after exposure to DE [39]. Since Garshong et al. [70] found the American dog tick nymph was not repelled by DE, this increased locomotor activity, in theory, should increase the DE exposure of LHTs when they encounter a treated area. Non-nidicolous ixodid ticks in nature undergo periods of questing where they are up on vegetation seeking for a host and non-questing when they are down beneath the leaf litter, moving up and down the vegetation [74]. The ascension and descension of the vegetation should expose ticks to DE on the surface of leaf litter. To test this hypothesis, we treated pine needle leaf litter infested with unfed LHT nymphs and found 100% mortality after 24 h when the litter was topically treated by the drop spreading of DE powder at the rate of 5 g DE/m^2^. These results are encouraging, showing that DE could be used as an alternative to pyrethroids to control LHTs, with the advantage of being used for organically certified animal farming as well. DE is also a natural (mineral), non-chemical, minimum risk pesticide with essentially long shelf life with no re-entry restrictions after application. Further making the case for future field studies for the use of DE to control LHTs, Showler and Harlien [42] found that a ground treatment with DE could be used to control the cattle fever tick, *Rhipicephalus microplus*, but could also prevent attachment and feeding on calves. The latter is significant in preventing or reducing pathogen transmission and, in both cases, point to the potential of using DE to control LHTs.

## 5. Conclusions

DE is efficacious in laboratory and semi-field studies against LHT nymphs, killing them within 24 h. DE does not affect the locomotor activity of LHTs except during the first few hours of dry powder application. The mode of action of DE against LHTs is unclear. Efforts should be directed towards field applications and determining the better mode of application (in terms of safety and efficacy)—whether applying DE in an aqueous medium by spraying or dry powder application. Although DE has a great potential to contribute towards the non-chemical means of controlling LHT populations, more research is still needed, such as field trials.

## Data Availability

The raw data generated during the experiment can be made available to interested parties with a reasonable timeline for the request.
